# Community composition of coral-associated Symbiodiniaceae differs across fine-scale environmental gradients in Kāne‘ohe Bay

**DOI:** 10.1098/rsos.212042

**Published:** 2022-09-07

**Authors:** Mariana Rocha de Souza, Carlo Caruso, Lupita Ruiz-Jones, Crawford Drury, Ruth Gates, Robert J. Toonen

**Affiliations:** ^1^ Hawai‘i Institute of Marine Biology, School of Ocean and Earth Science and Technology, University of Hawai‘i at Mānoa, Kāne'ohe, HI 96744, USA; ^2^ Chaminade University of Honolulu, 3140 Waialae Ave, Honolulu, HI 96816, USA

**Keywords:** Symbiodiniaceae, spatial pattern, coral reef

## Abstract

The survival of most reef-building corals is dependent upon a symbiosis between the coral and the community of Symbiodiniaceae. *Montipora capitata*, one of the main reef-building coral species in Hawai'i, is known to host a diversity of symbionts, but it remains unclear how they change spatially and whether environmental factors drive those changes. Here, we surveyed the Symbiodiniaceae community in 600 *M. capitata* colonies from 30 sites across Kāne'ohe Bay and tested for host specificity and environmental gradients driving spatial patterns of algal symbiont distribution. We found that the Symbiodiniaceae community differed markedly across sites, with *M. capitata* in the most open-ocean (northern) site hosting few or none of the genus *Durusdinium,* whereas individuals at other sites had a mix of *Durusdinium* and *Cladocopium*. Our study shows that the algal symbiont community composition responds to fine-scale differences in environmental gradients; depth and temperature variability were the most significant predictor of Symbiodiniaceae community, although environmental factors measured in the study explained only about 20% of observed variation. Identifying and mapping Symbiodiniaceae community distribution at multiple scales is an important step in advancing our understanding of algal symbiont diversity, distribution and evolution and the potential responses of corals to future environmental change.

## 1. Introduction

Coral reefs are among the most biologically diverse and productive ecosystems on Earth and provide valuable ecosystem services as sources of tourism, coastal protection, natural products and nutrition [1–3]. The symbiotic interaction with an exceptionally diverse dinoflagellate (family Symbiodiniaceae) is inherently linked to the health and success of reef-building corals because they provide a large proportion of the coral energy requirement [4–7]. There are 11 described genera [8] of Symbiodiniaceae (and possibly many more yet to be described), each with different physiological characteristics that impact the nutrient provisioning and thermal tolerance of the coral host [9–16]. *Cladocopium* (previously *Symbiodinium* clade C) and *Durusdinium* (previously clade D) are the two genera most commonly hosted by corals in the Pacific [17]. *Cladocopium* is a generalist symbiont and also the most speciose genus [18,19] while *Durusdinium* is usually found in shallow corals exposed to elevated light, sea surface temperature or areas with high temperature variability [20] and is associated with increased resilience to thermal stress [9,16,19–22].

Thermal stress is the main threat affecting corals worldwide [23–27]. Sea temperatures in many tropical regions have increased by almost 1°C over the past 100 years and are currently increasing at approximately 1–2°C per century [27–30]. Temperature stress disrupts coral-dinoflagellate symbiosis, leading to algal symbiont loss and consequent paling, a phenomenon known as coral bleaching [31,32]. Mass coral bleaching events are increasing in frequency and duration, resulting in significant losses of live coral in many parts of the world [27,33–35]. Coral susceptibility to heat stress and bleaching is dependent on a wide range of factors, including the algal symbiont community they host [9,22,24,32,36]. Bleaching may also represent an opportunity for corals to rapidly change their current algal symbiont community composition to more resilient types [37,38]. However, this Adaptive Bleaching Hypothesis remains controversial because many coral taxa are algal symbiotic specialists (but see [39]), hosting a single algal symbiont taxon and although corals can sometimes change their Symbiodiniaceae symbionts, many also recover to the same algal symbiont community they had prior to bleaching [40–44], although a few studies have reported that corals were able to maintain the symbionts even after 2 years [45]. Furthermore, hosting the stress-tolerant *Durusdinium* often comes at an energetic cost, as it decreases the growth and metabolite exchange rate of the host [46–52].

Symbiodiniaceae assemblage structure in corals tends to be shaped by many factors, including the host species [18,53], large-scale factors like geography [54,55], and local scale factors like depth [36,56], habitat [21], and environmental factors such as light [57] and temperature [21,55]. Here, we investigated the local-scale environmental drivers of Symbiodiniaceae assemblage structure in a common reef-building coral *Montipora capitata*, which is one of the most abundant corals in Kāne‘ohe Bay and may harbour a community of *Cladocopium* (C), *Durusdinium* (D) or both symbiont genera [58–60]. Previous studies of the species in Hawai‘i have reported associations with C31 [58,61,62], C17, C21 [61,62] and *Durusdinium glynnii* (formerly, *Symbiodinium glynii* [ITS2 Type D1^61^,^62^] and D 4-6 [58]).

Here, we used high-throughput sequencing of the internal transcribed spacer region (ITS2) to identify the Symbiodiniaceae assemblage and the local-scale environmental drivers of symbiont community composition for 600 colonies of the common reef-building coral *Montipora capitata* collected from across 30 sites in Kāne‘ohe Bay. This fine-scale sampling of symbionts from corals across Kāne‘ohe Bay included the more environmentally extreme northern and southern regions not sampled by previous studies. Our comprehensive sampling provides a baseline for symbiont communities across the full environmental gradient of the bay against which future consequences of coral bleaching can be compared and allows us to investigate the role of the environmental gradient in shaping the symbiont community of *Montipora capitata*.

## 2. Material and methods

### 2.1. Site selection and tagging

Details of the stratified random sampling design and environmental data are detailed by Caruso *et al*. [63]. Briefly, the bay was divided into five hydrodynamically defined regions (blocks) from South to North based on the water flow regimes and water residence time [64,65] and six sites were selected within each block following a stratified random sampling design [63]. Within each of these six sites, 20 visually healthy *M. capitata* were tagged, for a total of 600 tagged colonies distributed across 30 sites spread equally across the five hydrodynamically defined blocks. In early 2018, about 1 cm^2^ was sampled from each of 600 tagged *Montipora capitata* colonies. Sampled fragments were immediately preserved in 70% ethanol and stored at −20°C until processed. DNA was extracted using the Nucleospin Tissue Kits (Macherey-Nagel, Düren, Germany) following manufacturer instructions and quantified by fluorimetry (Quant-it HS dsDNA kit, Thermo-Fisher). Only corals that appeared healthy were sampled for this study.

**Figure 1.**
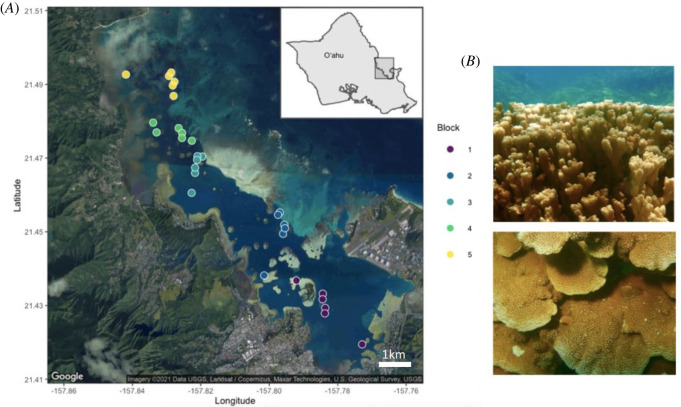
(*A*) Sites and blocks in Kāne‘ohe Bay. Each point is a randomly selected site within blocks represented by colours (Site IDs in electronic supplementary material, table S1). Blocks go from the south bay (block 1) to north bay (block 5). (*B*) *Montipora capitata*, the rice coral. The figures illustrate the morphological plasticity of this species, which can be branching (top) or plating (lower picture). Site IDs consist of the digit corresponding to the block in which the site is contained, followed by the site number (e.g. 1_10, with six sites per block, but not necessarily in consecutive order). Map done in the R package ggmap [67].

Data loggers (Hobo Pendant or Water Temp Pro v. 2 loggers, Onset Computer Corp., Bourne, MA) recorded temperature and sediment traps were collected regularly from each site [63] and used to calculate variation in temperature and sedimentation rate (standard deviation, maximum, minimum and range for each). Degree heating weeks (DHW) per site was calculated as the number of weeks when temperature exceeded the bleaching threshold of 28.5°C (MMM +1°C; [59,66]) (figure 1).

### 2.2. Symbiodiniaceae ITS2 amplicon sequencing library preparation

Symbiodiniaceae amplicon library preparation and sequencing followed the protocol outlined in [68]. Briefly, the ITS2 region of Symbiodiniaceae ribosomal DNA was targeted for sequencing using Symbiodiniaceae primers 454-ITSinfor2 (5′-GAATTGCAGAACTCCGTG-3′), ITSD (5′-GTGAATTGCAGAACTCCGTG-3′) and 454-ITS2-reverse (5′-GGGATCCATATGCTTAAGTTCAGCGGGT-3′) and its2rev2 (5′-CCTCCGCTTACTTATATGCTT-3′) modified from Arif *et al.* [69] to include Nextera indexes to allow multiplexing (see electronic supplementary material). Library products were sequenced on the Illumina MiSeq platform (v. 3 2 × 300 bp PE). Each library plate included a negative control (wells to which no template DNA was added). In addition, to ensure repeatability and test for the effect of sequencing run on the detection of symbionts, 192 samples (two 96 well plates) were sequenced more than one time in different runs. We found no differences in either community composition or symbiont identity that would alter our results or interpretation, and so the replicated control samples were simply pooled and analysed as a single sample for that individual.

### 2.3. Amplicon sequencing analysis with symportal

Raw sequences were first demultiplexed using Cutadapt [70]. Demultiplexed forward and reverse reads were submitted to SymPortal [71], a platform for genetically identifying Symbiodiniaceae using high throughput ITS2 sequence data that differentiates intra- and intergenomic sources of *ITS2* sequence variance (electronic supplementary material, figure S1).

Many terms have been used to describe the Symbiodiniaceae unity of resolution. To avoid ambiguity, we restrict our use to ‘Symbiodiniaceae type’ and ‘Symbiodiniaceae profile’. A type refers to Symbiodiniaceae taxa that have a specific sequence as their most abundant sequence. A Symbiodiniaceae profile is a set of ITS2 sequences that have been found in a sufficient number of independent samples to be identified as a ‘defining intragenomic variant’ (DIV). For example, C17 is a Symbiodiniaceae type and C17d/C31-C21-C17e-C21ac-C17f-C17 g is a Symbiodiniaceae profile with C17d present in higher abundance than the other types.

### 2.4. Statistical analysis

All analyses were done in the R statistical environment [72]. Bray-Curtis dissimilarity of relative abundance of the Symbiodiniaceae community composition was tested by permutational multivariate analysis of variance (PERMANOVA) in the function *adonis* (for effect of block, with site nested within block), and pairwise.*adonis* (for pairwise PERMANOVA) in the vegan package [73], each with 999 permutations. To better visualize the similarity among blocks, *R*^2^ from the PERMANOVA was plotted in a dendrogram using the function *pheatmap* in the package gplots [74]. The function *metaMDS* was used in the R package *vegan* [73] to generate non-metric multidimensional scaling visualizations using Bray-Curtis dissimilarities of algal symbiont community per block.

To investigate the effect of the environmental data in driving the Symbiodiniaceae community, we performed distance-based redundancy analysis (dbRDA) using the function *capscale*. After running an ANOVA to check the significance of the constrains axis as well as the significance of the environmental variables (electronic supplementary material, table S1), we visualized the significant variables in the dbRDA. Samples were considered to have majority *Cladocopium* (C) or *Durusdinium* (D) if the proportion of either algal symbiont exceeded 80% in the sample (modified from [60]). All remaining samples with *Cladocopium* and *Durusdinium* were designated as CD, corresponding to corals with neither algal symbiont genus achieving greater than 80% dominance. Correlation among the environmental factors was calculated using the function *cor.test* in R. Data and R code to execute and reproduce all the analyses and figures presented in the manuscript are archived at Zenodo [75].

## 3. Results

### 3.1. Symbiodiniaceae ITS2 sequences and ITS2 type profiles

550 *Montipora capitata* samples returned high-quality reads. Prior to quality filtering, these samples included 3 285 481 sequences which were reduced to 1 632 505 following quality control and minimum entropy decomposition with SymPortal, an average of 2968 sequences per sample. 11 386 sequences had not been previously curated by SymPortal and were unique to this dataset. A total of 283 Symbiodiniacae types were identified, 85% belonging to the genus *Cladocopium*, and 15% belonging to the genus *Durusdinium*. Twenty-six ITS2 type profiles were identified across all samples, 23 of which were from the genus *Cladocopium*, with the remaining three belonging to the genus *Durusdinium*. Overall, 43% of *Montipora capitata* hosted *Cladocopium* only, 11% hosted *Durusdinium* only, and 46% hosted a combination of both genera. From those mixed colonies, 32.5% were dominated by *Cladocopium* (C greater than 80%), while 37% of colonies were dominated by *Durusdinium* (D > 80%). To confirm differences in read number do not change the results or interpretation, we excluded 20 samples whose number of reads were more than two standard deviations above or below the mean.

### 3.2. Biogeography of symbiodiniaceae in Kāne‘ohe Bay

**Figure 2.**
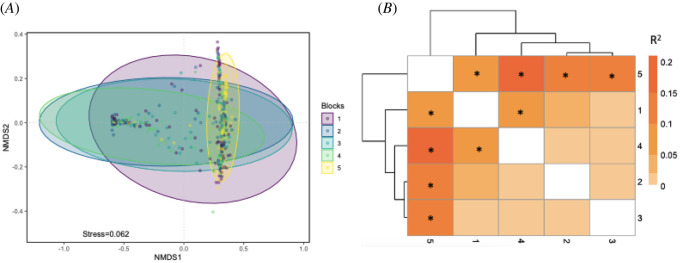
Similarity of Symbiodiniaceae communities detected in 600 *Montipora capitata* colonies collected across five hydrodynamically defined blocks in Kāne‘ohe Bay (block 1 is furthest south, while block 5 is furthest north in the bay). (*A*) nMDS of Symbiodiniaceae per block. Each point represents the symbiont community in a colony. Ellipses are 95% confidence intervals. (*B*) Dendrogram and heat map of *R*^2^ of the PERMANOVA of Symbiodiniaceae per block highlighting the similarity among blocks in the center of the bay that have been sampled in previous studies relative to block 1 and 5.

Symbiodiniaceae composition varied significantly among corals within each of the environmentally delineated blocks (PERMANOVA, *F*_25_ = 6.5632, *p* = 0.001; electronic supplementary material, table S2). Pairwise comparisons revealed that blocks 1 and 5 were significantly different, whereas blocks 2–4 were rarely significantly different from one another (electronic supplementary material, table S2, figure 2*a,b*).

**Figure 3.**
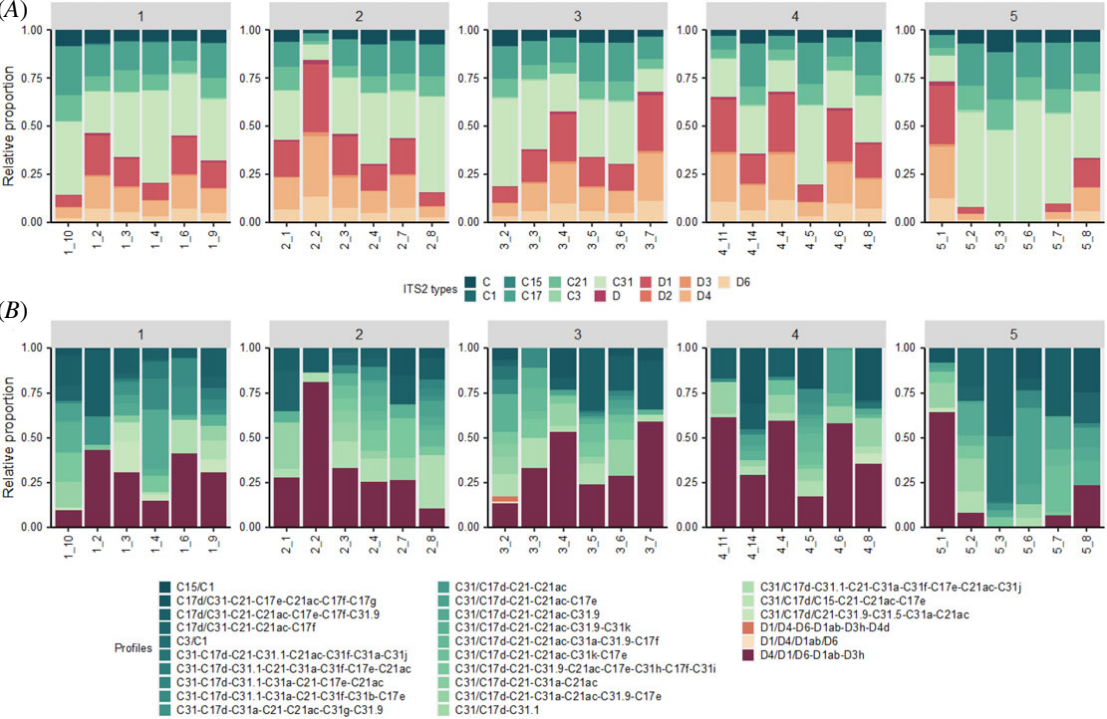
(*A*) Major Symbiodiniaceae types by site and block. (*B*) Symbiodiniaceae profiles by site. Block 1 is the southernmost in Kāne‘ohe Bay, while block 5 is the most northern and similar to offshore conditions. Symbiodiniaceae ITS2 subtypes were summarized to the major subtype to facilitate visualization in the bar charts (i.e. C31a and C31b were summarized as C31). Due to the wide diversity of ITS2 available in the SymPortal database, not all sequences are given names. Only those sequences that are used in the definition of ITS2 type profiles (i.e. DIVs) are named. Unnamed *Cladocopium* and *Durusdinium* sequences were combined for visualization and represented as summed ‘C’ and ‘D’ types, respectively.

Most sites had corals with both *Cladocopium* and *Durusdinium* present, except two sites in block 5 (sites 5_3 and 5_6) which did not have any samples in which *Durusdinium* was detected (figure 3*a*,*b*; electronic supplementary material, figure S1). It is noteworthy that sites 5_3 and 5_6 were also the least diverse of all we sampled, and unlike other sites, some colonies hosted only a single type (C3) of algal symbiont (electronic supplementary material, figure S1). Corals within block 5 hosted significantly less *Durusdinium* symbionts than any other site, with all sites including corals hosting a majority of *Cladocopium*. Site 2_2 had the highest relative proportion of *Durusdinium* and site 3_2 was the only site to have three profiles of *Durusdinium*.

### 3.3. Environmental drivers of symbiodiniaceae community composition

**Figure 4.**
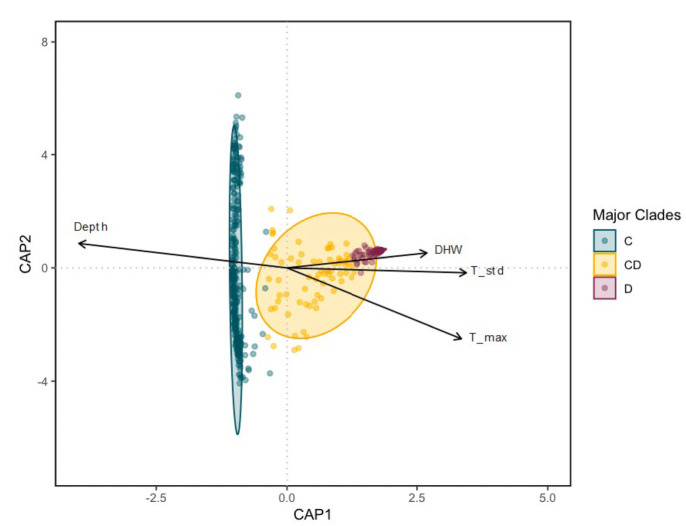
Distance based redundancy analysis (dbRDA) for environmental drivers of the Symbiodiniaceae communities in *Montipora capitata* sampled throughout Kāne‘ohe Bay. Each point represents an individual colony sampled irrespective of site. Samples were considered as majority *Cladocopium* (C) if they contain greater than 80%C, and majority *Durusdinium* (D) if greater than 80% D. Only vectors for the environmental factors contributing significantly to the algal symbiont diversity are plotted. Each arrow signifies the multiple partial correlation of the environmental driver in the RDA whose length and direction can be interpreted as indicative of its contribution to the proportion of variation explained.

Environmental data from each site is provided in Supplemental materials (electronic supplementary material, tables S1 & S3). dbRDA showed that *M. capitata* hosting majority *Cladocopium* are distinct from *M. capitata* hosting majority *Durusdinium* or mixed C & D (figure 4). PERMANOVA of the environmental factors in the dbRDA (electronic supplementary material, table S4) showed that depth, DHW, maximum mean daily temperature recorded at the site and mean daily standard deviation were all significant drivers of Symbiodiniaceae community composition. The environmental factors in the study explained only 20% of the Symbiodiniaceae variation, with depth having the greatest relative contribution (61%) followed by daily temperature standard deviation (19%), maximum temperature (9.9%) and DHW (4.8%) (electronic supplementary material, table S4).

## 4. Discussion

Here, we contribute to a more nuanced understanding of coral algal symbiosis by examining the associations of environmental drivers across a small spatial scale (approx. 10 km) on the community composition of Symbiodiniaceae in one of the dominant reef-building corals in the region, *Montipora capitata*. Previous surveys of the Symbiodiniaceae community structure of *M. capitata* across Kāne‘ohe Bay [60,61] found algal symbiont structure differed at the level of site and colony, but did not find significant differences in community composition of symbionts among regions of the bay. Here we report significant differences in symbiont community composition across the environmental gradient of Kāne‘ohe Bay that is not seen in these previous surveys. This difference may result from the increased resolution possible with high-throughput methods in our study. Stat *et al*. [61] used cloning and Sanger sequencing of ITS2 to identify algal symbionts, with fewer total colonies sampled (52), each with far less resolution (5–7 clones per colony) and in a smaller portion of the bay (Blocks 1 and 2) than is possible with the amplicon approach using high-throughput sequencing employed here. Innis *et al*. [60]) sampled a large number of colonies (707) using a qPCR assay targeting *Cladocopium* and *Durusdinium* but did not have the resolution to identify the diversity of subtypes of *Cladocopium* and *Durusdinium* as in this study. However, it is not the additional resolution so much as the geographic range of sampling that appears to underlie the difference, because Innis *et al*. [60] had no samples from either the far north or far south of the bay (blocks 1 & 5 in our study). Innis *et al*. [60] concluded that Symbiodiniaceae community variability may arise from either holobiont phenotypic plasticity or differential survival of Symbiodiniaceae across light gradients and recommended additional study into whether algal symbiont communities were stable or plastic within individuals across these gradients. Our findings are concordant with both Stat *et al*. [61] and Innes *et al*. [76] in that there is little differentiation among symbiont communities across the central portion of Kāne‘ohe Bay (blocks 2–4), but without blocks 1 and 5, in which we found the greatest difference in symbiont composition, they did not sample the ends of the environmental gradient over which the community composition is observed to shift significantly in our study.

Using a high-throughput metabarcoding approach, our study shows that the Symbiodiniaceae community composition varied at all spatial scales examined, both among the sites within each region and among hydrodynamically defined blocks across the bay. Interestingly, the relative proportion of algal symbiont types is relatively consistent among the colonies (i.e. when *Cladocopium* is present, C31 is typically most abundant followed by C17 and C21, whereas for *Durusdinium*, D1 is the most abundant, followed by D4 and D6), no matter the location (figure 3; electronic supplementary material, figure S1). In total, seven types of *Cladocopium* (C1, C3, C15, C17, C21, C31) are detected among colonies across Kāne‘ohe Bay, with C31, C17 and C1 being the most common. Similarly, we detected six types of *Durusdinium* (D, D1, D2, D3, D4, D6) with D1, D4, and D6 being the most prevalent. These results are consistent with multiple studies reporting strong Symbiodiniaceae specificity with type C31 being the most common symbiont associated with *Montipora capitata* in Hawai‘i [58,61,62], but the distinctly structured communities found in the northern and southern extremes of the bay were undetected by previous studies.

Despite considerable variation in the composition of the communities of Symbiodiniaceae hosted by *Montipora capitata* sampled across Kāne‘ohe Bay, we find that the coral–algal symbiont association was strongly influenced by environmental gradients. *M. capitata* located at the environmental extremes (block 1 in the south and block 5 in the north) hosted Symbiodiniaceae communities that were significantly different from blocks in the centre of the bay. We find four factors (depth, DHW, maximum and variability in temperature) that are significant drivers of Symbiodiniaceae community composition (electronic supplementary material, table S4), although these four significant factors combined explained less than 20% of the variation in symbiont community composition. Although 80% of the variation remains unexplained, meta-analyses show that environment generally explains about 20% of the variation in community structure across terrestrial, freshwater and marine ecosystems [77]. Other factors such as light [57], nutrients [78,79] and pH [80] were unmeasured here, but have also been found to influence the symbiont community in other systems. It is important to note that in our study, we cannot determine if these correlated environmental factors are the direct drivers of algal symbiont diversity. Until future studies determine the underlying mechanisms driving variation in algal symbiont community composition, we remain cautious in our interpretation of these environmental drivers.

### 4.1. Depth

Consistent with previous studies [18,57,60,81,82], depth appears to be the strongest environmental driver of symbiont community composition measured in this study. The deepest block (5) hosted the lowest proportion of *Durusdinium*, with most colonies hosting only *Cladocopium* (figure 3; electronic supplementary material, figure S1). A similar pattern is found when looking at individual sites surveyed across all blocks, with the deepest sites (1_3, 1_4, 1_9, 1_10, 2_4 and 2_8) being mostly dominated by *Cladocopium*. While Innis *et al*. [60] also found depth to be the primary driver of algal symbiont clades in Kāne‘ohe Bay they concluded the mechanism was most likely light attenuation in deeper reefs as opposed to depth *per se*. It is well known that light attenuates with depth in water, but the relationship is complicated in coral reef environments where a variety of other factors can alter penetration of light to deeper corals [83–85]. Likewise, an entire suite of environmental parameters other than light also covary with depth, such that it is often difficult to know exactly which environmental or biological factors drive changes in community structure with depth [86–89]. Depth is simple to measure and is well-correlated with changes in coral reef community structure in many studies (reviewed by [86,90,91]) but is much harder to isolate as an environmental factor to determine the quantitative relative contribution in studies such as this (electronic supplementary material, table S5).

### 4.2. Sedimentation

Suspended sediments can both impact corals directly and alter their light penetration and irradiance. Sedimentation can also impact corals negatively through increased nutrient input, damaging the coral surface and by making it harder for corals to feed and photosynthesize [92–94]. Sediment particles in the water increase turbidity, which attenuates light similarly as does depth. In our study, however, none of the sediment parameters were significant predictors of symbiont community composition (electronic supplementary material, table S4). The lack of significance may result from the complex interaction of turbidity and depth on light because deeper low sediment reefs (like most sites in block 5) may experience higher irradiance than shallower sites with higher turbidity.

### 4.3. Temperature

The role of temperature in mediating the symbiosis between the coral host and their algal endosymbionts has been widely studied [31,32,95,96], so it is not surprising that several aspects of temperature come out as significant drivers of community composition. Degree heating weeks (DHW) and maximum temperature are widely established to be major predictors of the breakdown of symbiosis between the partners and result in coral bleaching [27,97]. Unsurprisingly, both were significant in our analysis of environmental drivers, although neither explained much of the variation in algal symbiont community structure among sites. By contrast, variability in temperature, in this case, mean daily temperature standard deviation, had the second largest contribution to the observed variation in algal symbiont community structure. A number of studies highlight the importance of daily temperature fluctuations for coral acclimatization to higher temperatures [98,99], suggesting that temperature fluctuations encourage greater thermal tolerance by exposing the corals to short periods of thermal stress without causing mortality. Blocks in the centre of the bay had higher temperature variation (higher mean average temperature standard deviation and higher mean daily range) and our data shows that the existing community structure of algal symbionts responds to such variability (figure 3). Blocks in the centre of the bay (blocks 2–4) had higher proportions of *Durusdinium*, while block 1 and block 5 (extreme north and south, respectively) had the lowest proportion, suggesting that blocks at the extreme ends of the bay may be more vulnerable to bleaching events. This prediction is consistent with coral surveys during the 2015 bleaching event in Kāne‘ohe Bay which found the highest levels of bleaching and paling in the north (70%) and south (60%) of the bay [100].

Here we advance previous work by showing that algal symbiont communities within a single species of coral in a single embayment are finely tuned to their environmental conditions. Whether community response results from selection for Symbiodiniaceae types living under different environments, adaptive shuffling of Symbiodiniaceae communities in response to environmental conditions, or both, remains to be determined. Comparing microscale environmental variability [101,102] to algal symbiont community structure might explain much of the variability we see, because algal symbionts in colonies may be adaptively responding to fine-scale variability at the same site within the broad regional differences we compared here. A more detailed understanding of the relationship between adaptive tuning of algal symbiont communities to local environmental conditions will require fine-scale environmental measurements coupled with long-term monitoring of corals in the field to determine whether and how algal symbiont communities within individual colonies respond through time.

## 5. Conclusion

Fine-scale sampling of 600 *M. capitata* colonies across a relatively small spatial gradient (approx. 10 km) within Kāne‘ohe Bay showed that algal symbiont community structure is associated with depth and temperature. This fine-scale variation in algal symbiont community composition across local environmental gradients suggests that algal symbiont communities can adaptively match the environmental conditions surrounding the holobiont. Previous studies of Symbiodiniaceae in the Bay focused on the three central regions that exhibit the least environmental variability among sites across the environmental gradient from north to south. Here we extend the sampling to include the full gradient across the bay and overturned previous conclusions that algal symbiont structure did not differ significantly among regions of the bay. Our results support that conclusion for symbiont communities in the central portion of the bay, but show that both environmental extremes of the far northern and southern regions of Kāne‘ohe Bay sampled here for the first time differ significantly from those in the central region. These results imply that the community composition responds to the conditions under which the holobiont is living, setting the stage for understanding the role of environmental conditions in how Symbiodiniaceae communities are distributed in space and time.

Our study highlights the complex interactions among environmental factors and algal symbiont diversity in the reef-building coral *Montipora capitata*. While depth was the main factor driving algal symbiont community composition in our study, several aspects of temperature (DHW, max temp & mean standard deviation) likewise appear to be drivers of algal symbiont community composition. We also note that many factors correlate with depth, such as light, temperature, sedimentation rate and water flow, such that fine-scale measurements of the full range of environmental factors surrounding individual colonies through time will be needed to pinpoint the most important environmental drivers of algal symbiont community structure. Regardless of the ultimate suite of parameters that drive algal symbiont community structure in corals, our study shows that Symbiodiniaceae communities are attuned to fine-scale environmental gradients and that understanding these complex interactions across the heterogeneous mosaic of coral reef environments is needed to better predict spatial patterns in biological responses such as bleaching susceptibility.

## Data Availability

All materials, code and data are available for download on Zenodo (75; https://zenodo.org/record/5670832#.YvBKpC1h2iM). The data are provided in electronic supplementary material [103].
